# Clinical and Molecular Characterization of a Rare Case of BNT162b2 mRNA COVID-19 Vaccine-Associated Myositis

**DOI:** 10.3390/vaccines10071135

**Published:** 2022-07-16

**Authors:** Eli Magen, Sumit Mukherjee, Mahua Bhattacharya, Rajesh Detroja, Eugene Merzon, Idan Blum, Alejandro Livoff, Mark Shlapobersky, Gideon Baum, Ran Talisman, Evgenia Cherniavsky, Amir Dori, Milana Frenkel-Morgenstern

**Affiliations:** 1 Medicine C Department, Clinical Immunology and Allergy Division, Barzilai University Medical Center, Ben-Gurion University of the Negev, Ashkelon 7830604, Israel; iblum2000@gmail.com; 2 Leumit Health Services, Tel Aviv 6473817, Israel; emerzon@gmail.com; 3 Azrieli Faculty of Medicine, Bar-Ilan University, Safed 1311502, Israel; sumit.mukherjee@biu.ac.il (S.M.); mahua.bhattacharya@biu.ac.il (M.B.); rajesh.detroja@biu.ac.il (R.D.); gidi.baum2@gmail.com (G.B.); 4 Department of Computer Science, Ben-Gurion University, Beer-Sheva 8410501, Israel; 5 Department of Family Medicine, Sackler Faculty of Medicine, Tel Aviv University, Tel Aviv 6997801, Israel; 6 Pathology Department, Barzilai University Medical Center, Ashkelon 7830604, Israel; alejandrol@bmc.gov.il (A.L.); marks@bmc.gov.il (M.S.); 7 Plastic Surgery Department, Barzilai University Medical Center, Ashkelon 7830604, Israel; rant@bmc.gov.il; 8 Imaging Department, Barzilai University Medical Center, Ashkelon 7830604, Israel; evgeniac@bmc.gov.il; 9 Department of Neurology, Sheba Medical Center, Ramat Gan 5262000, Israel; amir.dori@sheba.health.gov.il; 10 Sackler Faculty of Medicine, Tel Aviv University, Tel Aviv 6997801, Israel

**Keywords:** BNT162b2, mRNA vaccine, COVID-19, myositis

## Abstract

Initial clinical trials and surveillance data have shown that the most commonly administered BNT162b2 COVID-19 mRNA vaccine is effective and safe. However, several cases of mRNA vaccine-induced mild to moderate adverse events were recently reported. Here, we report a rare case of myositis after injection of the first dose of BNT162b2 COVID-19 mRNA vaccine into the left deltoid muscle of a 34-year-old, previously healthy woman who presented progressive proximal muscle weakness, progressive dysphagia, and dyspnea with respiratory failure. One month after vaccination, BNT162b2 vaccine mRNA expression was detected in a tissue biopsy of the right deltoid and quadriceps muscles. We propose this case as a rare example of COVID-19 mRNA vaccine-induced myositis. This study comprehensively characterizes the clinical and molecular features of BNT162b2 mRNA vaccine-associated myositis in which the patient was severely affected.

## 1. Introduction

The Food and Drug Administration (FDA) granted emergency approval for the Pfizer-BioNTech BNT162b2 mRNA COVID-19 vaccine to combat the COVID-19 pandemic [1] on 11 December 2020. In Israel, a nationwide COVID-19 vaccination campaign began in late December 2020, in which more than five million people were successfully vaccinated, leading to a rapid decline in COVID-19 cases throughout the country [2]. In phase III clinical trials, the BNT162b2 vaccine was found to be 95% effective against COVID-19 [3]. However, the safety and risk of adverse reactions to the BNT162b2 vaccine are now the major concerns. In the clinical trials, participants reported short-term mild local adverse reactions, including injection site pain, swelling, fever, fatigue, myalgia, and lymphadenopathy [4]. The occurrence of adverse events was found significantly to be higher in the younger populations than in older people and in females than in males [4,5,6]. The majority of adverse events in the younger population is one of the important causes behind COVID-19 vaccine hesitancy among youth [7].

Several cases of mild autoimmune reactions followed by BNT162b2 vaccine administration were reported where the requirement for hospital care was rare [8,9]. However, recent studies showed that in autoimmune inflammatory rheumatic diseases (AIIRD), the BNTb262 vaccine could generate immunogenic response in the majority of patients, which raised safety concerns for patients with AIIRDs [10,11,12]. Some evidence demonstrated that BNT162b2 could induce the development of Guillain-Barre syndrome (GBS), which is a rare neurological autoimmune disorder of the peripheral nervous system [13,14,15,16,17]. Furthermore, a recent case study reported that the BNT162b2 vaccine induced the development of acute transverse myelitis followed by fatal neurological complications [18]. A few cases have been reported for BNT162b2 vaccine-induced myasthenia gravis (MG) development, a chronic autoimmune condition affecting the neuromuscular junction [19,20,21]. However, some studies demonstrated that BNT162b2 vaccination showed overall good short-term safety in MG patients [22,23].

Recently, several mild to moderate rare cases of anaphylaxis [24,25,26,27], thrombocytopenia [28,29,30,31], myocarditis [32,33,34,35,36], myositis [37,38,39,40,41], and rhabdomyolysis [42,43,44] associated with the BNT162b2 vaccine have been reported. Here, we report a rare case of BNT162b2 vaccine-associated myositis in which the patient was severely affected. To understand the molecular mechanism behind the severe conditions of myositis, we analyzed the vaccine mRNA expression in the DNA and RNA samples from patient blood and muscle tissue biopsy.

## 2. Materials and Methods

### 2.1. Ethical Considerations

Written informed consent for publication was obtained from the patient. The authors assure the accuracy and comprehensiveness of the data in this report.

### 2.2. Immunoassays and Serological Testing

Nasopharyngeal swabs were taken and examined for SARS-CoV-2 by real-time RT-PCR performed with internal positive and negative controls, according to World Health Organization (WHO) guidelines. The COBAS SARS-CoV-2 6800/8800 assay (Roche Pharmaceuticals, Basel, Switzerland) was employed. SARS-CoV-2 IgG II quantitative testing was performed on the Abbott Alinity i platform.

### 2.3. DNA and RNA Extraction and Sequencing

A blood sample was collected from the patient 7 days after administration of the first vaccine dose. As a control, a blood sample was taken 7 days after the second vaccine dose from a second individual. Total RNA was extracted from peripheral blood mononuclear cells (PBMCs) using a RNeasy Mini Kit (Qiagen, Germany, catalogue no. 74104) according to the manufacturer’s protocol. FFPE (formalin-fixed paraffin-embedded) tissue sections were processed for total RNA and genomic DNA using a RNeasy FFPE kit (Qiagen, Germany, catalogue no. 73504) and a DNeasy Blood and Tissue kit (Qiagen, Germany, catalogue no. 69504), according to the manufacturer’s protocols. All samples were sent for RNA and DNA sequencing on an Illumina NextSeq 550 machine.

### 2.4. Nested PCR Assay for BNT162b2 Vaccine

A PCR was performed for 20 cycles with the outside primers, after which the primers were removed with a MN Nucleospin PCR and Gel Clean-up kit. A nested PCR was performed for 30 cycles, when the products were separated on a 3% agarose gel.

## 3. Results

### 3.1. Case Representation

A previously healthy 34-year-old woman with no evidence of prior SARS-CoV-2 infection presented with complaints of severe muscle weakness, pain, and tenderness. The patient denied vigorous exercise, seizures, or heavy physical labor before the onset of symptoms. Her symptoms began on day four after the first dose of BNT162b2 mRNA vaccine, which was administered into her left deltoid muscle. On admission, she had no fever, chills, or malaise. A COVID-19 swab (PCR) test was performed twice and was negative both times. She did not complain of cough or shortness of breath. Upon physical examination, the patient was afebrile and had a blood pressure of 118/72 mmHg, a pulse rate of 84 b.p.m., and oxygen saturation of 97% on room air. There was remarkable swelling, severe tenderness, and proximal muscle weakness in the flexor muscles of the neck, pelvic region, thigh and shoulders, with symmetrical distribution. A neurological examination showed intact higher mental function. Sensory and cranial nerve examination results were within normal limits. There was significant muscle weakness, with a strength grade of 3/5 for the shoulders and hips, and 2/5 for the elbows, ankles and wrists. All nerve reflexes were normal. A chest radiograph was normal, and an echocardiogram showed no pericardial effusion and good biventricular function.

Upon admission, a blood workup revealed a creatine kinase (CK) level of 15750 IU/L (*n* < 195 IU/L), an aspartate aminotransaminase (AST) level of 351 U/L, an alanine aminotransaminase (ALT) level of 138 U/L, and a C-reactive protein (CRP) level of 134 mg/L (*n* < 5 mg/L), as well as a normal hemoglobin level, leukocyte count, and platelet count. The patient showed lymphopenia at 0.43 × 10^9^ cells/L (normal 1.5–4.5 × 10^9^ cells/L), and her high-sensitivity troponin T (hsTnT) level was 12 ng/L (normal < 14 ng/L). Antinuclear antibody tested using indirect immunofluorescence on human type 2 epithelial cells (Hep2) was strongly positive with a speckled pattern. Testing for anti-double-stranded DNA, anti-SSA/SSB, anti-Sm, anti-melanoma differentiation-associated gene 5 (anti-MDA5), anti-Jo1, anti-Scl70, anti-Ro52, and anti-neutrophil cytoplasmic antibodies (ANCA), were all negative. Anti-acetylcholine receptor (AChR) and anti-muscle-specific tyrosine kinase (MuSK) autoantibodies were negative. Anti-TIF-1g, NXP-2/MJ and Mi-2 antibodies were not available in our clinical laboratory.

High-resolution computed tomography (CT) of the lungs showed bilateral mild pleural effusion. Influenza PCR and HIV, EBV, CMV, HBV, HCV, and parvovirus serological results were all negative. Urinalysis excluded myoglobinuria. Eight weeks after one dose of BNT162b2 vaccine, the SARS-CoV-2 IgG level was 37 AU/L (Alinity i System, Abbott Laboratories, IL; a value < 50 AU/mL is considered negative, a value > 150 AU/mL is considered protective, and a value between 50 and 150 AU/mL reflects the uncertainty of protection).

**Figure 1:**
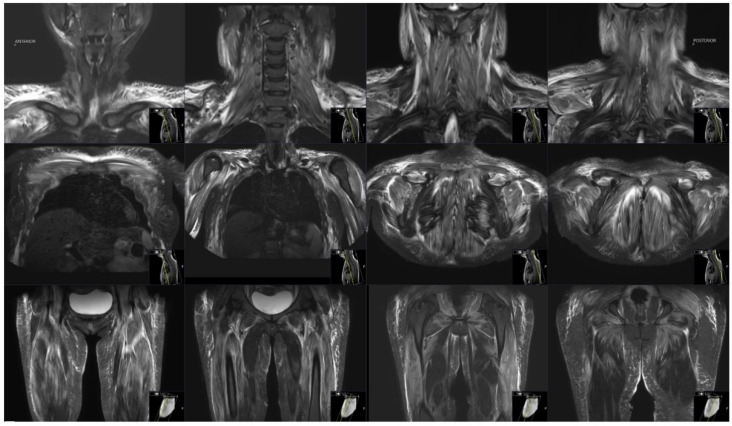
MRI of deltoid and thigh muscles. Bilateral patchy and diffuse areas of high T2/ STIR signal intensity in muscles are seen, consistent with active myositis.

Electromyography and nerve conduction studies performed three weeks after disease onset showed evidence of myositis with muscle fiber denervation confined to the proximal upper limbs (specifically to the deltoid muscle). There was no electrodiagnostic evidence for large fiber polyneuropathy or lumbosacral radiculopathy. Magnetic resonance imaging (MRI) of the deltoid and thigh muscles was suggestive of myositis with bilateral symmetric involvement (Figure 1). No other polymyositis-associated features, including specific heliotrope or Gottron’s signs or papules, other skin or cuticular changes, Raynaud’s phenomenon, lymphadenopathy, arthritis, or cardiac involvement were noted. A diagnosis of myositis was made on the basis of characteristic proximal muscle weakness, elevated creatine phosphokinase (CPK) levels, a strongly positive ANA HEp2, and electrodiagnostic evidence for active myositis with precise MRI findings.

**Figure 2:**
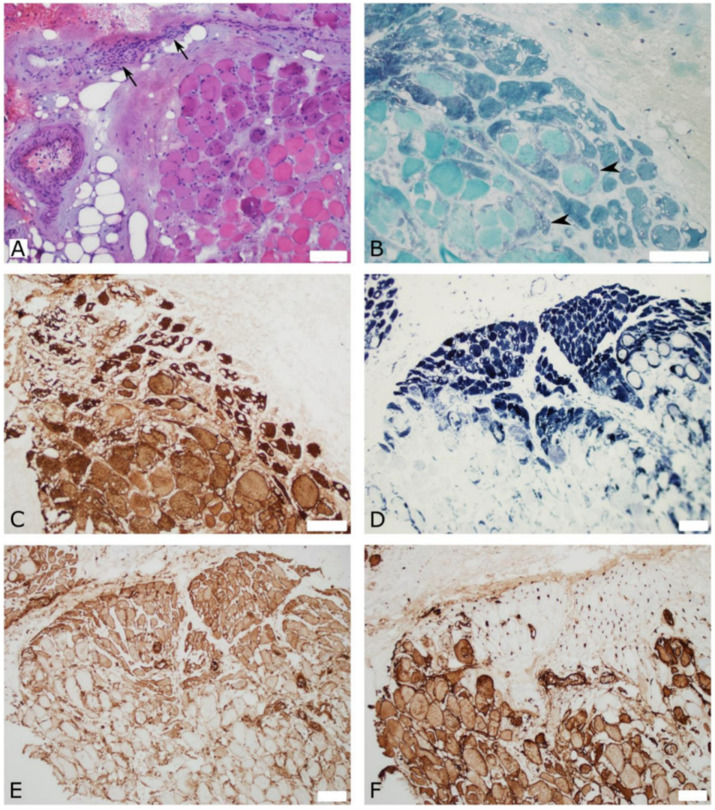
Quadriceps muscle biopsy with perifascicular findings that are suggestive of myositis. (**A**) Hematoxylin and eosin stain shows perivascular mononuclear cellularity (arrows) in the perimysium, and perimysial and endomysial fibrosis. (**B**) Gomori trichome stain shows perifascicular atrophic fibers and small vacuoles, as well as signs of regeneration (arrowheads). (**C**) Slow myosin immunostaining highlights perifascicular atrophy and small vacuoles. (**D**) Dense staining with reduced nicotinamide adenine dinucleotide (NADH) dehydrogenase in perifascicular atrophic fibers, and no staining of necrotic fibers. (**E**) Major histocompatibility complex class I (MHC-I) is up-regulated in perifascicular myofibers. (**F**) Membranolytic attack complex (C5b-9) immunostaining shows significant capillary deposition in the perifascicular region and highlights necrotic fibers. Scale bars = 100 μm.

Open muscle biopsies from the right deltoid and quadriceps performed one month after vaccination showed similar lesions in both muscles that included perivascular inflammation, perifascicular fiber atrophy with vacuoles, major histocompatibility complex class I (MHC-I) staining, and C5b9 complement deposition in the capillaries. Other regions showed diffuse fiber necrosis suggestive of muscle infarction. These findings were most consistent with a diagnosis of myositis (Figure 2).

The patient was treated with intravenous methylprednisolone (125 mg b.i.d. as a bolus) for ten days, followed by oral prednisone (1 mg/kg qd). Subsequently, there was a transient ~50% improvement in proximal muscle weakness, with the patient’s serum CPK being monitored daily as it continued to rise. On day 15, the patient developed progressive dysphagia, dyspnea with hypoxemia, and hypercapnia requiring invasive mechanical ventilation and nasogastric feeding. She was resuscitated and transferred to the intensive care unit. Pulmonary embolism was ruled out by CT angiography. The patient was treated with methylprednisolone pulse therapy (1000 mg daily) and intravenous immune globulin (IVIG), which resulted in mild improvement. Although partial bulbar weakness persisted, the patient was able to resume and continue oral prednisone and azathioprine. After multiple failures to wean the patient from mechanical ventilation, a tracheostomy was performed. She is now undergoing respiratory and neurological rehabilitation, albeit without further improvement.

### 3.2. Evaluation of Vaccine mRNA Expression in Blood and Muscle Tissue Biopsy Samples

**Figure 3:**
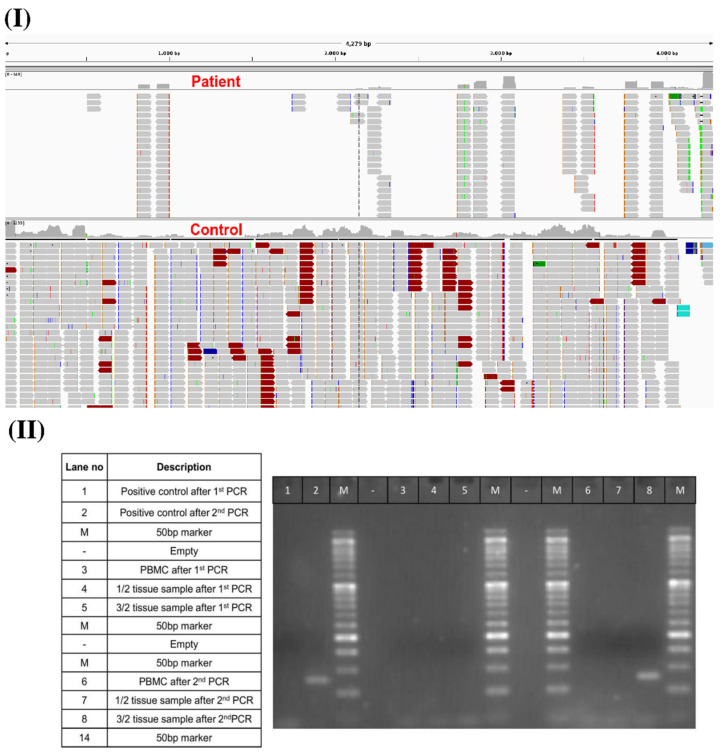
(**I**) Distribution of mapped RNA-seq reads across spike mRNA. (**II**) PCR validation of vaccine spike mRNA expression in the patient’s muscle tissue biopsy samples one-month post-vaccination.

To understand the association of BNT162b2 mRNA expression with the development of myositis, we sequenced the patient’s blood and muscle tissue biopsy samples. After generating raw sequence reads from control and patient blood samples, quality control was performed, followed by the removal of index and adapter sequences. Next, trimmed reads were further used for mapping to the human reference sequences + BNT162b2 vaccine spike mRNA using Bowtie2 [45]. Then, total mapped reads to the vaccine spike mRNA sequence region were calculated. A total of 17,626 and 639 reads were mapped to the vaccine spike protein mRNA sequence in the RNA-seq data of the control and the patient, respectively. Upon visualizing the spike mRNA reads mapping region using the Integrative Genomics Viewer (IGV) [46], we found that in the control sample, reads covered and were equally mapped across 98% region of the vaccine spike protein mRNA sequence. In the patient sample, reads were mapped to just a few regions covering only 36% of the vaccine spike protein mRNA sequence (Figure 3I). Partial mapping of the vaccine spike protein mRNA sequence in the patient’s sample indicated an unusual pattern of vaccine mRNA expression in blood cells, namely, “chopped” parts of the mRNA vaccine molecules from the Pfizer vaccine. This was supported by the low level of anti-SARS-CoV-2 IgGs detected, suggesting that the mRNA vaccine was not translated into the spike protein in this patient, resulting in no immune response to SARS-CoV-2.

Next, to understand the development of vaccine-induced myositis, we performed a DNA sequencing analysis of a right quadriceps muscle biopsy sample from the patient one-month post-vaccination. We did not find any mapped reads of the vaccine spike mRNA sequence in the genomic DNA sequencing data. It can thus be inferred that the vaccine mRNA sequences did not integrate into the patient’s genome. Next, to check whether vaccine mRNA expression could be detected in the RNA of the tissue biopsy sample, we performed a nested PCR using two sets of primers against the 3′UTR (untranslated region) of the vaccine mRNA. A synthetic construct containing the 3′UTR of the BNT162b2 mRNA vaccine served as a control for PCR validation of the mRNA vaccine. To achieve maximum sensitivity and specificity in PCR in efforts to detect the presence of vaccine mRNA in the quadriceps muscle tissue, a nested PCR was developed in which the forward primers annealed to the TLE5 3′UTR sequence and the reverse primers annealed to the mito-nc sequence of the BNT162b2 vaccine sequence. The expected size of the vaccine mRNA sequence was thus 75 bp. We observed a band of this size in the total tissue RNA, confirming the expression of the BNT162b2 vaccine mRNA in the biopsy samples of the right quadriceps muscle one month after BNT162b2 vaccination (Figure 3II). This result highlights that although the BNT162b2 vaccine mRNA was not properly expressed in blood cells seven days after receipt of the first vaccine dose, it was still expressed in muscle tissue distant from the vaccination site one month after receipt of the first vaccine dose. This suggests that the unusual BNT162b2 mRNA expression pattern observed in muscle cells may be related to the development of myositis.

## 4. Discussion

In this study, we represent a rare example of BNT162b2 vaccine-induced myositis. According to mRNA vaccine technology, most endogenous mRNA transcripts are rapidly degraded, usually within 10–15 min after inoculation [47]. The rate of degradation of synthetic mRNA vaccines in tissues is inversely proportional to the kinetics of translation initiation and duration. Therefore, to increase the stability of vaccine mRNA, it is necessary to optimize codon usage and UTR sequences [48]. The BNT162b2 mRNA vaccine is a lipid nanoparticle-encapsulated vaccine, with the nanoparticles protecting the mRNA from degradation by mediating endocytosis and endosomal escape [49]. After injection into the muscle, the synthetically produced BNT162b2 mRNA vaccine should be degraded by both extracellular and intracellular RNases, thus remaining in human tissues for only a few days [50]. However, in our patient, we observed the expression of vaccine mRNA in muscle tissues as late as one month after vaccination, which may indicate that the exogenously expressed mRNA was stable enough to persist over a long period of time. One of the known autoimmune manifestations of COVID-19-induced muscle disease is myositis with severe bulbar weakness [51]. Most commonly, SARS-CoV-2-associated muscle inflammation is triggered by clonal expansion of T cells and production of pro-inflammatory cytokines, leading to muscle damage [52,53].

Although BNT162b2 COVID-19 vaccination is safe and most of the adverse effects are mild [54], some moderate to serious adverse events such as anaphylaxis [24,25,26,27], thrombocytopenia [28,29,30,31], myocarditis [32,33,34,35,36], myositis [37,38,39,40,41] and rhabdomyolysis [42,43,44] were recently reported. Therefore, it is crucial to identify the factors associated with adverse effects after BNT162b2 vaccination. A direct relationship between the reduced BNT162b2-induced immunogenicity and the risk of autoimmunity has yet to be demonstrated. Thus, the case presented here raises many difficult questions and unanswered concerns, such as the efficacy and safety of mRNA vaccines in subgroups previously excluded from BNT162b2 vaccine trials and underscores the need for further studies in order to understand autoimmunity induced by modified-mRNA vaccines.

As the new COVID-19 strains are continuously emerging [55,56] and, in several countries, the COVID-19 surge shows a seasonal pattern [57,58], the vaccination strategy should be planned accordingly. The new clinical trials should be designed to understand the effect of BNT162b2 vaccination in groups of different autoimmune disorder patients, which could help to design an effective strategy for COVID-19 vaccination with minimal risk of adverse reactions. At present, considering the global scenario for effectiveness and risk of the COVID-19 vaccination, it could be suggested that the development of different strategies [59,60,61,62,63,64] with proper clinical trials is needed to design such new COVID-19 vaccines which could be effective against all SARS-CoV-2 variants with a minimal risk of adverse effects.

## Data Availability

Not applicable.
